# Evaluation of Fracture Strength of Ceramics Containing Small Surface Defects Introduced by Focused Ion Beam

**DOI:** 10.3390/ma11030457

**Published:** 2018-03-20

**Authors:** Nanako Sato, Koji Takahashi

**Affiliations:** 1 Graduate School of Engineering, Yokohama National University, 79-5 Tokiwadai, Hodogaya, Yokohama 240-8501, Japan; sato-nanako-nw@ynu.jp; 2 Faculty of Engineering, Yokohama National University, 79-5 Tokiwadai, Hodogaya, Yokohama 240-8501, Japan

**Keywords:** Al_2_O_3_/SiC, focused ion beam, surface defect, fracture strength, process zone size failure criterion, $\sqrt{area}$ parameter model

## Abstract

The aim of this study was to clarify the effects of micro surface defects introduced by the focused ion beam (FIB) technique on the fracture strength of ceramics. Three-point bending tests on alumina-silicon carbide (Al_2_O_3_/SiC) ceramic composites containing crack-like surface defects introduced by FIB were carried out. A surface defect with $\sqrt{area}$ in the range 19 to 35 µm was introduced at the center of each specimen. Test results showed that the fracture strengths of the FIB-defect specimens depended on $\sqrt{area}$. The test results were evaluated using the evaluation equation of fracture strength based on the process zone size failure criterion and the $\sqrt{area}$ parameter model. The experimental results indicate that FIB-induced defects can be used as small initial cracks for the fracture strength evaluation of ceramics. Moreover, the proposed equation was useful for the fracture strength evaluation of ceramics containing micro surface defects introduced by FIB.

## 1. Introduction

Ceramics have excellent mechanical, tribological, and thermal properties in comparison with metallic materials. However, there is a risk that failure initiates from defects due to the low fracture toughness of ceramics. Therefore, it is very important to quantitatively evaluate the influence of defects on fracture strength for maintenance management of ceramic components.

It is reported that the fracture toughness for small flaws is lower than that for large cracks. Thus, conventional linear elastic fracture mechanics (LEFM) is not applicable for predicting the fracture strength of ceramics containing small flaws [1]. Many authors have proposed various models for the relationship between defect size and fracture strength [2,3,4,5,6]. Ando et al. proposed the process zone size failure criterion and reported that the fracture strength of ceramics containing small surface cracks could be evaluated by this criterion [7]. This model can predict the fracture strength of a material having a semicircular crack introduced perpendicular to the tensile stress. Murakami and Endo proposed the $\sqrt{area}$ parameter model to predict the fatigue limit of metals having a surface crack with arbitrary shape [8]. Here, the $\sqrt{area}$ is the square root of the area of a surface defect projected onto the direction of the maximum tensile stress. The $\sqrt{area}$ parameter model is widely applied to evaluate the fatigue limit of metals [9,10]. In a previous study, we proposed a new evaluation equation for the fracture strength of ceramics combining the process zone size failure criterion and the $\sqrt{area}$ parameter model [11]. This equation is useful for the evaluation of fracture strength of ceramics having a surface crack with arbitrary shape and orientation introduced by the indentation method.

However, it is known that tensile residual stress is generated around the indentation when a pre-crack is introduced on the surface of a ceramic material by the indentation method [12]. Therefore, elimination of the tensile residual stress by surface polishing or annealing in inert gas is necessary [13]. If the tensile residual stress is not eliminated, the influence of residual stress on the fracture strength should be considered to predict the fracture strength with high accuracy [13]. The dimensions of a defect at the origin of a fracture in an actual ceramic material are equivalent to the grain size, which is less than 20 µm in a typical ceramic. However, the dimensions of defects introduced in most of the previous studies were larger than 20 µm because it is difficult to introduce small surface cracks by the indentation method [14,15,16].

In this study, we introduced crack-like surface defects on the surface of ceramics by the focused ion beam (FIB) technique. The FIB technique is a high-precision working method that uses the effect of sputtering by ion beam scanning on the surface of a material. Sakamoto et al. reported that the fatigue limits of FIB-notched specimens were equivalent to the fatigue limits of annealed fatigue-cracked specimens of carbon steel [17]. Rokio and Solin evaluated small crack initiation and propagation from small FIB-defect in high strength steel and reported that a FIB notch to initiate small cracks is a useful way to test small crack growth in high strength steels [18]. However, to our knowledge, the influence of FIB-introduced surface defects on the fracture strength of ceramics has not yet been studied. In this paper, the effects of microscopic surface defects introduced by FIB on the fracture strength of an Al_2_O_3_/SiC composite are clarified. Regarding the relationship between fracture strength and defect size, the consistencies of the test results and the values predicted by the proposed evaluation equation were verified.

## 2. Evaluation Equation of Fracture Strength

In this section, an outline of the proposed equation for fracture strength evaluation is described [11]. The equation is based on the process zone size failure criterion [7] and the $\sqrt{area}$ parameter model [8,19].

Ando et al. proposed the following equation to predict the fracture strength (*σ*_C_) of ceramics with a small surface crack focusing on the process zone size at the crack tip and [7]

(1) $$\frac{\pi }{8}{\left(\frac{K_{\mathrm{IC}}}{\sigma_{0}}\right)}^{2}=a_{e}\left\{\sec\left(\frac{\pi \sigma_{C}}{2\sigma_{0}}\right)-1\right\},$$

where *K*_IC_ (MPa·m^1/2^) is the fracture toughness, and *σ*_0_ (MPa) is the fracture strength of a smooth specimen assuming a defect is sufficiently small, and *σ*_C_ (MPa) is the fracture strength of a specimen containing a surface crack. The equivalent crack length, *a*_e_ (µm) is defined as

(2) $$a_{e}=\frac{1}{\pi }{\left(\frac{K_{\mathrm{IC}}}{\sigma_{c}}\right)}^{2},$$

On the other hand, Murakami showed that the maximum stress intensity factor (*K*_max_) at the edge of a surface crack with arbitrary shape under tensile stress (*σ*_∞_) could be approximated within 10% error by [19]

(3) $$K_{\max}≅0.650\sigma_{\infty }\sqrt{\pi \sqrt{area}},$$

where $\sqrt{area}$ is the square root of the area of a surface defect projected onto the direction of the maximum tensile stress. When *σ*_C_ is applied to a surface crack, the *K*_max_ value reaches *K*_IC_. Equation (3) leads to

(4) $$K_{\mathrm{IC}}=0.650\sigma_{C}\sqrt{\pi \sqrt{area}}\;.$$

The relationship between fracture strength *σ*_C_ and the crack size $\sqrt{area}$ is expressed as follows based on Equations (1), (2) and (4)

(5) $$\sigma_{C}=\cos^{-1}\left(\frac{3.38\sigma_{0}{}^{2}\sqrt{area}}{\pi \cdot K_{\mathrm{IC}}{}^{2}+3.38\sigma_{0}{}^{2}\sqrt{area}}\right)\cdot \frac{2\sigma_{0}}{\pi }\;.$$

**Figure 1:**
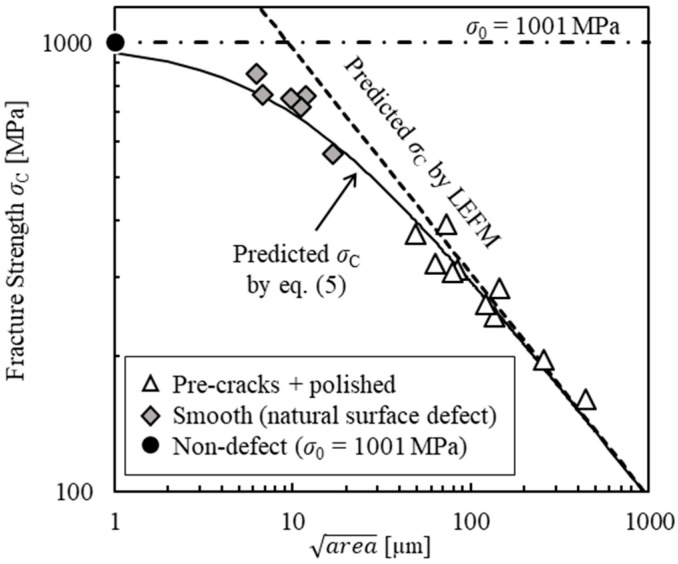
Relationship between fracture strength and $\sqrt{area}$ of Al_2_O_3_/SiC [11].

This is the equation to evaluate the fracture strength of a ceramic material containing a surface defect with arbitrary shape. Figure 1 shows the relationship between *σ*_C_ and $\sqrt{area}$ of alumina reinforced by silicon carbide (Al_2_O_3_/SiC) [11]. The dotted line indicates the *σ*_C_ predicted based on conventional LEFM. The predicted values of *σ*_C_ based on LEFM are overestimate for $\sqrt{area}$ less than 20 µm. Thus, conventional LEFM is not applicable to ceramics with small cracks. On the other hand, the solid line indicates the *σ*_C_ predicted based on Equation (5). Although the *σ*_C_ predicted based on Equation (5) were slightly smaller than those of the experimental results, they agreed relatively well with the experimental results in the range of 5–20 µm. In this study, the relationship between $\sqrt{area}$ of an FIB defect and fracture strength was evaluated using *K*_IC_ and *σ*_0_ as the basic mechanical properties of the sample material.

## 3. Experimental Procedures

### 3.1. Test Specimen

Alumina reinforced by silicon carbide (Al_2_O_3_/SiC) was selected as a test material in this study. The Al_2_O_3_ powder (AKP-50, Sumitomo Chemical Industry Co. Ltd., Tokyo, Japan) used in this study had a mean particle size of 0.2 µm. The SiC powder (ultrafine grade, Ibiden Co. Ltd., Gifu, Japan) used had a mean particle size of 0.27 µm. The samples were prepared using a mixture of Al_2_O_3_ with 15 vol % SiC powder. To this mixture, isopropanol was added, and the mixture was blended thoroughly for 24 h by ball milling. After drying and comminution, the mixture was hot-pressed at 1973 K and 35 MPa for 2 h in an N_2_ atmosphere [20]. The hot-pressed plates were cut into bending test specimens measuring 3 × 4 × 20 mm. The surface of each specimen was polished to a mirror-like finish. 

### 3.2. Specimen Preparations

To heal surface cracks induced during machining, all the bending test specimens were heat-treated at 1573 K for 1 h in air, which are the optimum healing conditions for surface cracks in Al_2_O_3_/SiC [20]. These specimens are called “smooth” specimens. The *σ*_0_ value was evaluated by the strength distribution of the smooth specimens.

**Figure 2:**
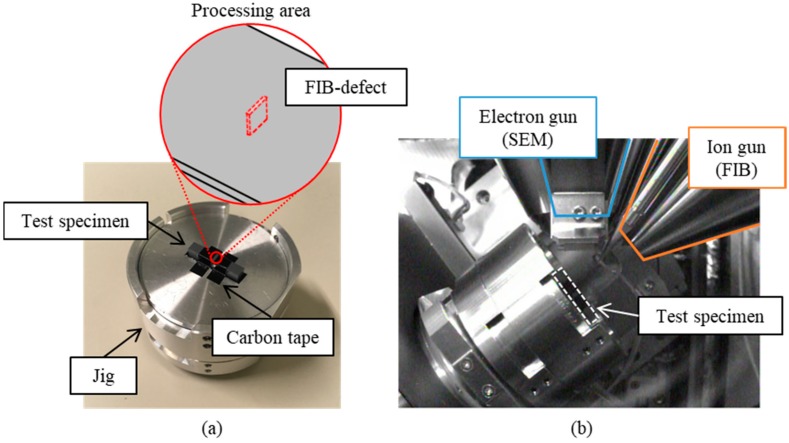
Photographs of the FIB process: (**a**) fixing method of test specimen; (**b**) inside of device.

**Figure 3:**
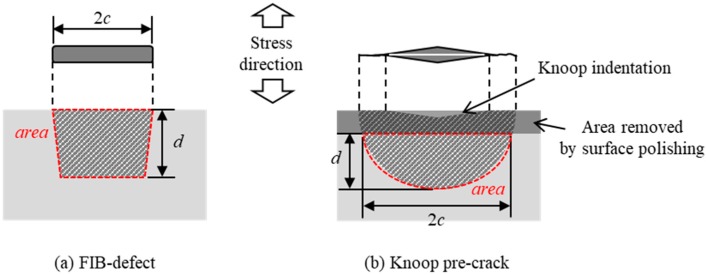
Schematic illustrations of surface defects: (**a**) FIB-defect specimen; (**b**) pre-crack + polished specimen.

An FIB defect was introduced at the center of each smooth specimen by using a multi beam SEM-FIB system JIB-4501 (by JEOL Ltd., Tokyo, Japan). These specimens are called the “FIB-defect” specimens. Figure 2 shows the detail of the FIB technique process. A test specimen was fixed on a jig with carbon tape. The FIB defect was processed while monitoring the processing state using the SEM. Figure 3a schematically shows the shape of an FIB defect. The surface lengths (2*c*) ranged from 20 to 60 µm and the depths (*d*) ranged from 19 to 24 µm. The conditions of the FIB technique are presented in Table 1. The area of a defect was evaluated based on the fracture surface observation.

For comparison, “pre-crack + polished” specimens were prepared in the following procedure. First, a Knoop indentation was made at the center of each smooth specimen. Figure 3b shows the shape of a Knoop pre-crack. The indentation loads ranged from 9.8 to 49 N. Next, these specimens were polished using a 1-µm diamond slurry to eliminate the tensile residual stress around the indentations. The amount of material removed from the surface was 4 to 6 times the indentation depth. The ASTM standard C1421 suggests removing 4.5 to 5 times the indentation depth [21]. Thus, the removed depth in this study almost met the standard.

### 3.3. Measurement of Fracture Toughness

The fracture toughness *K*_IC_ value was evaluated by the indentation fracture (IF) method. Vickers indentations were made on the polished surface of a bending test specimen by the application of an indentation load of 49 N for 15 s. Then, *K*_IC_ (Pa·m^1/2^) was calculated using the following equation in accordance with the Japan Industrial Standards [22]

(6) $$K_{\mathrm{IC}}=0.026\frac{E^{\frac{1}{2}}P^{\frac{1}{2}}a}{c^{\frac{3}{2}}},$$

where *E* (Pa) is the Young’s modulus, *P* (N) is the indentation load, *a* (m) is half the diagonal length of the indentation, and *c* (m) is half the surface crack length. The units of calculated values of *K*_IC_ were converted into “MPa·m^1/2^”. Crack length was measured using an optical microscope with total magnifications of 580× and higher to avoid the measurement error of *K*_IC_ values [23].

### 3.4. Measurement of Fracture Strength

**Figure 4:**
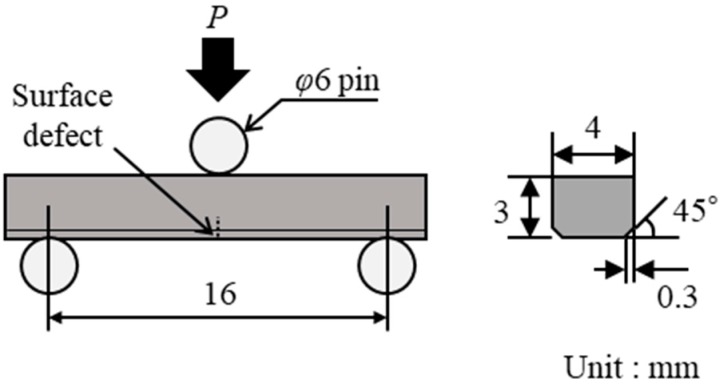
Schematic diagram of the three-point loading system and size and dimensions of test specimen.

The *σ*_C_ values of each specimen were measured by a three-point bending test, as shown in Figure 4, at room temperature in air. The span length was 16 mm, and the crosshead speed was 0.5 mm/min. After the bending test, the fracture surfaces were observed by laser microscopy, and the size of the defects at the fracture origins was measured.

## 4. Results and Evaluation of Fracture Strength

### 4.1. Mechanical Properties of the Sample Material

In this section, the calculated results of *K*_IC_ and *σ*_0_ are presented. An average and a standard deviation of the *K*_IC_ value of four samples measured by the IF method were 3.50 ± 0.05 MPa·m^1/2^. The scatter of the *K*_IC_ value is quite small.

**Figure 5:**
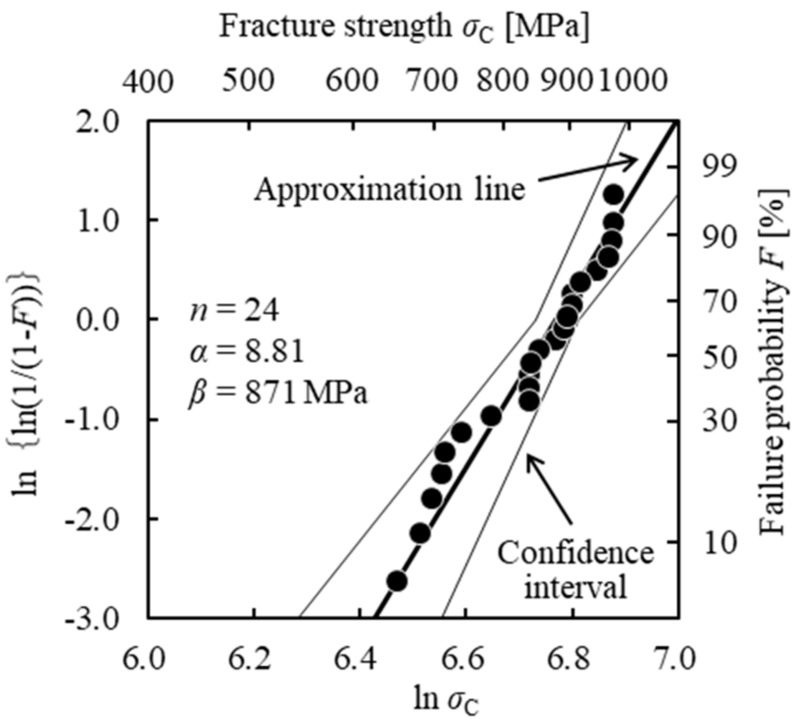
Two-parameter Weibull distribution of fracture strength of the smooth specimens.

The *σ*_0_ value of the sample material was estimated using the strength distribution of the smooth specimens. The two-parameter Weibull function is expressed by

(7) $$F\left(\sigma_{C}\right)=1-\exp\left\{-{\left(\frac{\sigma_{C}}{\beta }\right)}^{\alpha }\right\}\;,$$

where *F*(*σ*_C_) is the cumulative failure probability based on the median rank method, *α* is the scale parameter, and *β* is the shape parameter. Figure 5 shows the two-parameter Weibull distribution of the smooth specimens. The test results were plotted as the relationship between ln (*σ*_C_) and ln{ln(1/(1 − *F*)} led by Equation (7). The second horizontal and vertical axis show *σ*_C_ and *F* values, respectively. Two parameters were determined using the maximum likelihood method based on JIS R1625 [24]. The *α* value was 8.81, and the *β* value was 871 MPa. In Figure 5, a thick solid line and thin solid lines show the approximation line and the 90% confidence interval, respectively. The 90% confidence interval values of *α* and *β* are (*α*_L_, *α*_U_) = (6.72, 11.6), (*β*_L_, *β*_U_) = (838, 910). In the Weibull distribution, the *F*(*σ*_C_) value increased as the defect dimensions in the specimens decreased. Because the size of defects in a smooth the specimen with *F*(*σ*_C_) = 99% considered to be sufficiently small, the *σ*_C_ value at *F*(*σ*_C_) = 99% is regarded as *σ*_0_. This evaluation method of *σ*_0_ showed a good estimation results of *σ*_C_ as shown in Figure 1 [11]. Substituting the values of *α* and *β* of the smooth specimen and *F*(*σ*_C_) = 99% in Equation (7), *σ*_0_ = 1036 MPa was calculated.

### 4.2. Fracture Strength of FIB-Defect Specimens

**Figure 6:**
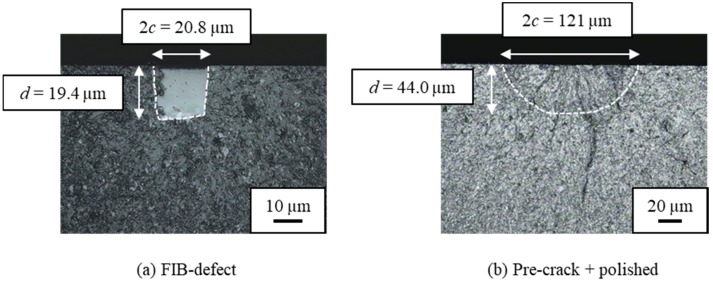
Laser microscope images of fracture surfaces: (**a**) FIB-defect specimen, $\sqrt{area}$ = 19.1 µm, *σ*_C_ = 687 MPa; (**b**) pre-crack + polished specimen, $\sqrt{area}$ = 64.6 µm, *σ*_C_ = 400 MPa.

All specimens containing FIB-induced surface defects fractured at the FIB-defect area as a result of the three-point bending tests. Figure 6a,b show laser microscope images of fractured surfaces of a FIB-defect specimen and a pre-crack + polished specimen, respectively. The surface length 2*c*, the depth *d*, and the $\sqrt{area}$ of the FIB-defects and pre-cracks after polishing were measured by these observations. Table 2 shows the results of the bending tests for the FIB-defect specimens and the pre-crack + polished specimens. The sizes of the FIB defects were in the following range: $\sqrt{area}$ = 19–35 µm. The *σ*_C_ value of the FIB-defect specimens decreased with increasing $\sqrt{area}$. Similar to the pre-crack + polished specimens, *σ*_C_ showed strong $\sqrt{area}$-dependency in the FIB-defect specimens.

### 4.3. Comparison Between Predicted Fracture Strength and Test Results

**Figure 7:**
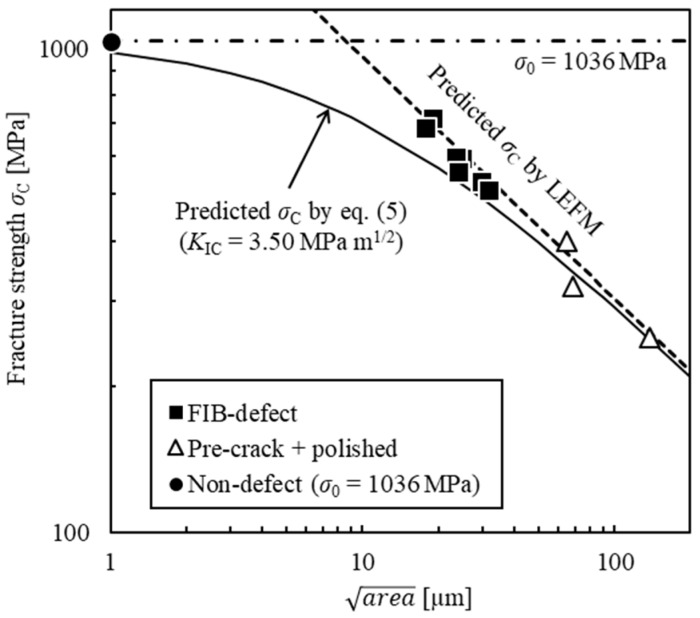
Comparison between the predicted fracture strength and the bending test results.

The *σ*_C_ of the sample material was predicted substituting *K*_IC_ = 3.50 MPa·m^1/2^ and *σ*_0_ = 1036 MPa into Equation (5). Figure 7 shows the predicted *σ*_C_ values and the bending test results. For the pre-crack + polished specimens, the predicted *σ*_C_ values matched the experimental results. Thus, the usefulness of this equation was confirmed. The errors between the experimental and predicted results were generally within ±15%. It is thought that the prediction errors are mainly caused by the approximation errors of *K*_max_ by Equation (3). For the FIB-defect specimens, the experimental *σ*_C_ are between the predicted *σ*_C_ based on LEFM and Equation (5). The predicted values of *σ*_C_ based on LEFM and Equation (5) give the upper and lower limit, respectively. Similar tendency that Equation (5) gives lower *σ*_C_ was observed in the results shown in Figure 1. The results indicate that a FIB-defect can be used as a small initial crack for fracture strength evaluation of ceramics. It was found that the evaluation equation was useful for evaluating the fracture strengths of ceramics containing micro surface defects introduced by FIB.

## 5. Conclusions

This study investigated the fracture strengths of Al_2_O_3_/SiC samples in which micro surface defects had been introduced by the FIB technique. The test results were evaluated using the proposed evaluation equation of fracture strength. The results are summarized as follows:

(1) The micro surface defects in the size range of $\sqrt{area}$ = 19–35 µm were introduced on the surface of Al_2_O_3_/SiC by the FIB technique.
(2) The fracture strengths of the FIB-defect specimens showed $\sqrt{area}$ dependency. This result is similar to that obtained for the pre-crack + polished specimens.
(3) FIB-defect can be used as a small initial crack for fracture strength evaluation of ceramics.
(4) The fracture strengths of the FIB-defect specimens predicted by the evaluation equation matched the test results. It was confirmed that the proposed equation for fracture strength evaluation was useful for ceramics containing micro surface defects introduced by FIB.

## Figures and Tables

**Table 1:** FIB processing conditions.

Ion Source	Ga Liquid Metal Ions
Accelerating voltage	30 kV
Prove current	3000 pA
Dose	190–280 nC/µm^2^

**Table 2:** Bending test results.

Type of Defect	Surface Length 2*c* (µm)	Depth *d* (µm)	$\sqrt{area}$ (µm)	Fracture Strength *σ*_C_ (Mpa)
FIB-defect test specimen	20.8	19.4	19.1	687
	20.0	23.2	20.4	715
	33.4	21.1	25.3	597
	32.3	22.5	26.2	557
	32.8	23.8	27.4	592
	46.2	24.1	32.6	530
	60.1	21.3	35.2	508
Pre-crack + polished test specimen	121	44.0	64.6	400
	124	47.5	68.0	322
	228	106	137	253
